# The Effect of Statin Therapy on Coronary Plaque Composition Using Virtual Histology Intravascular Ultrasound: A Meta-Analysis

**DOI:** 10.1371/journal.pone.0133433

**Published:** 2015-07-30

**Authors:** Guian Zheng, Yuxin Li, Huishan Huang, Jinghan Wang, Atsushi Hirayama, Jinxiu Lin

**Affiliations:** 1 The First Clinical Medical College of Fujian Medical University, Fuzhou, Fujian, China; 2 Department of Cardiology, Zhangzhou Hospital Affiliated to Fujian Medical University, Fujian, China; 3 Department of Advanced Cardiovascular Imaging Analysis, Nihon University School of Medicine, Tokyo, Japan; 4 Department of Molecular Biology, Zhangzhou Medical Science Institute, Fujian, China; 5 Department of Hematology, Zhejiang Institute of Hematology, First Affiliated Hospital of Zhejiang University School of Medicine, Hangzhou, China; 6 Department of Cardiology, Nihon University School of Medicine, Tokyo, Japan; 7 Department of Cardiology, First Affiliated Hospital of Fujian Medical University, Fujian, China; Medstar Washington Hospital Center, UNITED STATES

## Abstract

**Objective:**

Previous studies have indicated that statin therapy may promote plaque regression. However, the impact of statin therapy on plaque composition has not been clearly elucidated. We performed a meta-analysis to investigate the effect of statin therapy on coronary plaque composition as assessed by virtual histology intravascular ultrasound (VH-IVUS).

**Methods:**

Online databases were searched from inception to March 1, 2015. Studies providing VH-IVUS volumetric analyses of coronary plaque composition at baseline and follow-up in patients receiving statin therapy were included. Weighted mean difference (WMD) using a random-effects model was used.

**Results:**

Ten studies involving 682 patients were included. There was a substantial reduction in fibrous volume between baseline and follow-up (WMD: -2.37 mm^3^, 95% confidence interval (CI) -4.01 to -0.74 mm^3^, P=0.004), and a significant increase in dense calcium (DC) volume (WMD: 0.89 mm^3^, 95% CI 0.70 to 1.08 mm^3^, P<0.00001). No significant change was seen in fibro-fatty and necrotic core (NC) volumes. In stratified analyses, the fibrous volume was decreased significantly (WMD: -3.39 mm^3^, 95% CI -6.56 to -0.21 mm^3^, P=0.04) and the absolute DC volume (WMD: 0.99 mm^3^, 95% CI 0.23 to 1.76 mm^3^, P=0.01) was increased in the subgroup with ≥12 months follow-up, whereas no significant change was observed in the subgroup with < 12 months follow-up. Similarly, a substantial decrease in fibrous volume (WMD: -2.01 mm^3^, 95% CI -3.05 to -0.96 mm^3^, P< 0.0002) and an increase in DC volume (WMD: 0.90 mm^3^, 95% CI 0.70 to 1.10 mm^3^, P< 0.00001) were observed in the subgroup with high-intensive statin therapy, while the change in fibrous and DC volumes approached statistical significance (P=0.05 and P=0.05, respectively) in the subgroup with low-intensive statin therapy.

**Conclusions:**

Statin treatment, particularly of high-intensity and long-term duration, induced a marked modification in coronary plaque composition including a decrease in fibrous tissue and an increase in DC.

## Introduction

It is known that statin therapy may retard the progression of atherosclerosis, and even promote regression of atherosclerotic plaques [1–3]. Intravascular ultrasound (IVUS) with color mapping, such as virtual histology IVUS (VH-IVUS), provides an effective and reproducible method for evaluating plaque tissues, and enables a thorough qualitative and quantitative analysis of plaque composition [4]. Multiple studies have revealed that statin therapy may reduce plaque volume [3,5,6]. However, the effect of statin therapy on plaque composition remains unclear. A previous meta-analysis [7] pooled the data from two VH-IVUS trials [8,9] and suggested that statin therapy did not change plaque composition. However, in the Venus study, 6 months of atorvastatin induced a significant modification of plaque components [10]. Thus, the available data are conflicting.

In this study, we performed a meta-analysis to evaluate the impact of statin therapy on coronary plaque composition using VH-IVUS. Stratified analyses were also conducted to determine the influence of intensity and duration of statin therapy on plaque composition.

## Methods

### Search Strategy and Selection Criteria

We performed this meta-analysis in accordance with the Preferred Reporting Items for Systematic Reviews and Meta-Analyses (PRISMA) guidelines [11] (S1 Checklist). We searched PubMed, Embase, the Cochrane Library, and Web of Science from inception to March 1, 2015 without language restriction. The following were used as medical subject heading terms and/or keywords: “3-hydroxy-3-methylglutaryl coenzyme a reductase(s),” “statin(s),” “HMG-CoA reductase inhibitor(s),” “atorvastatin,” “pravastatin,” “simvastatin,” “cerivastatin,” “fluvastatin,” “lovastatin,” “mevastatin,” “pitavastatin,” “rosuvastatin,” and “intravascular ultrasound,” “intravascular ultrasonography,” “IVUS.” We also searched ClinicalTrials.gov for potentially relevant trials which were not identified in our electronic database search. The complete search strategy is available in the supporting information (S1 Table).

Our predefined inclusion criteria were as follows: (1) VH-IVUS volumetric analysis of coronary plaque composition at baseline and follow-up; (2) the volumetric outcomes were reported as absolute volume; (3) ≥ one group of patients receiving statin therapy; (4) clinical studies published in peer-reviewed journals.

Exclusion criteria were as follows: (1) studies analyzing stents, in-stent neointima, other drugs, statins plus other drugs; (2) studies without follow-up; (3) no volumetric outcomes of plaque composition; (4) not a VH-IVUS analysis; (5) reviews, case reports, meeting abstracts or gray literature.

### Data Extraction

Two authors (GZ and JW) independently confirmed the eligibility of studies and extracted the data from the qualifying studies according to the standard Cochrane protocol [12]. Discrepancies were resolved by consensus or a third reviewer if necessary. Data extracted from each qualifying trial included study design characteristics, number of patients, patients’ basic characteristics (age, sex), type and dose of statin, low-density lipoprotein cholesterol (LDL-C) levels, volumes of plaque and plaque composition at baseline and follow-up, and follow-up duration.

### Outcome Measures

All IVUS examinations were performed using auto-motorized pullback (at 0.5 mm/s) after intracoronary administration of nitroglycerin. VH-IVUS imaging was analyzed by independent experienced investigators who were blinded to the clinical presentation. The same segment was analyzed at baseline and at follow-up based on reproducible landmarks (i.e. side branch, calcifications, or unusual plaque shapes). VH-IVUS analysis classified plaques into four major compositions: fibrous, fibro-fatty (FF), dense calcium (DC), and necrotic core (NC). These different plaque compositions were assigned a color code of green, yellow-green, white and red, respectively. Each plaque composition was represented in absolute volume.

The primary endpoint of interest was the change in absolute volume of each plaque composition between baseline and follow-up in patients receiving statin therapy. Stratified analyses were also performed according to the intensity and duration of statin therapy. Based on the intensity of statin therapy, studies were grouped into the high-intensive statin therapy subgroup and the low-intensive statin therapy subgroup. High-intensive statin therapy was defined as atorvastatin 40–80 mg/day or rosuvastatin 20–40 mg/day [13]. The secondary endpoint was the change in absolute plaque volume between baseline and follow-up.

### Quality Assessment

For randomized controlled trials (RCTs), we assessed the risk of bias in the included studies using the quality assessment tool recommended in version 5.1.0 of the Cochrane Handbook for Systematic Reviews of Interventions [12]. For nonrandomized studies, we used the Newcastle–Ottawa scale to assess the quality of selected studies [14]. This scale uses a star system to evaluate observational studies based on three broad parameters: participant selection, comparability of study groups and ascertainment of outcome or exposure. The maximum score on this scale was 9 points. We defined a total score of 7 to 9 as high quality, a total score of 4–6 as medium, and a total score of <4 as low.

### Statistical analysis

All statistical analyses were conducted with Review Manager 5.2 (The Cochrane Collaboration, Oxford, UK). We calculated weighted mean differences (WMD) with 95% confidence interval (CI) from the mean and standard deviation (SD). When data were reported as median and range, we contacted the authors to request actual mean and SD. If data with mean and SD could not be obtained [15], we estimated the mean and SD according to appropriate formulas introduced by Hozo et al. [16]. A random-effects model was used to pool data. A P value < 0.05 was considered statistically significant. Potential sources of heterogeneity in studies were assessed using Cochran’s Q statistic and the I^2^ statistics. I^2^ <25% was regarded as minimal heterogeneity, 25%≤ I^2^ <50% as moderate, and I^2^ ≥50% as high. The possibility of publication bias was assessed using funnel plots. Egger’s test was performed when funnel plots were asymmetric.

## Results

### Study selection and characteristics

**Fig 1:**
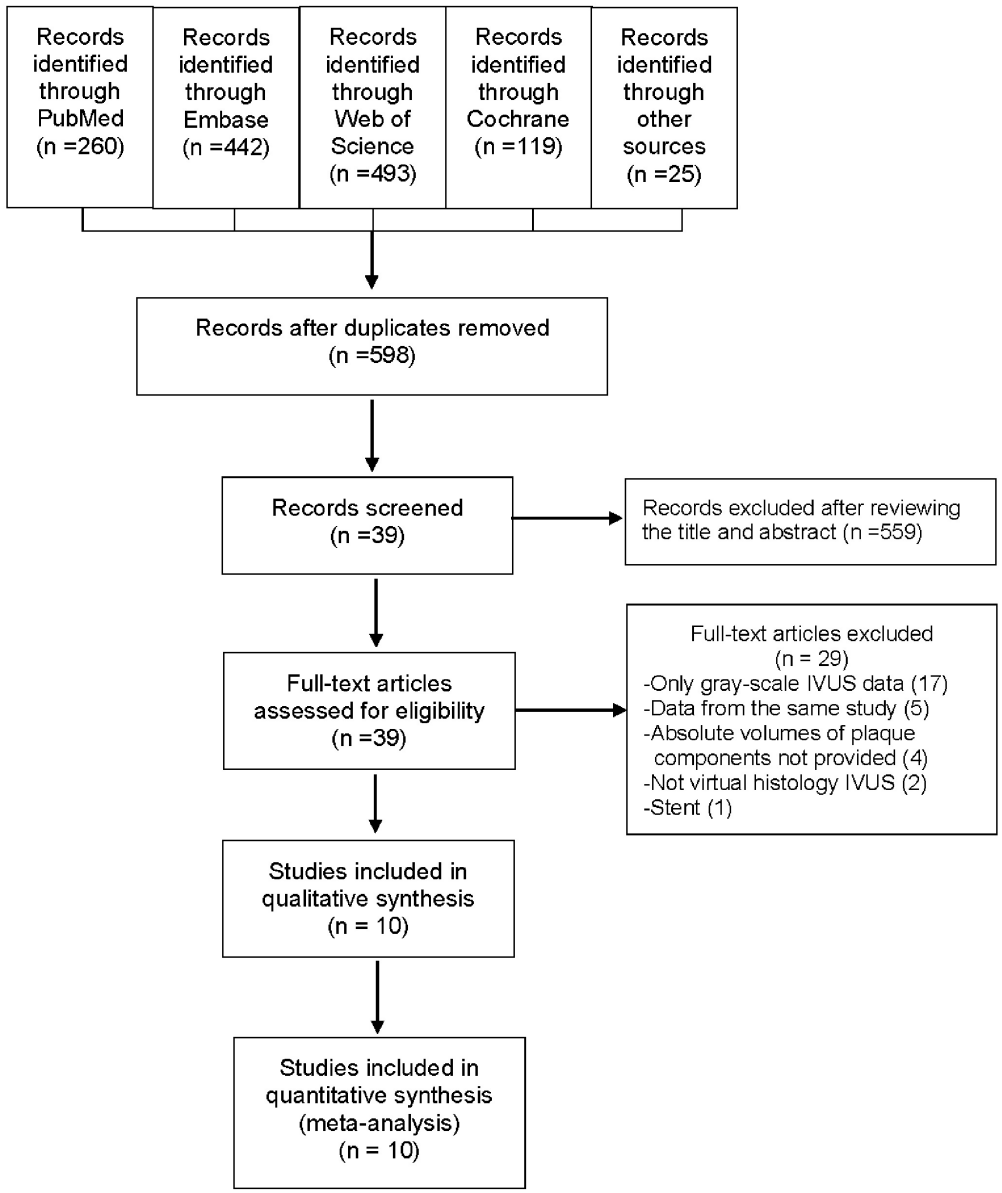
Flow diagram showing the selection of studies for the meta-analysis. Abbreviations: IVUS: intravascular ultrasound.

The literature search identified 1339 potentially eligible publications. We screened the titles and abstracts for inclusion after removing duplicates and 39 studies were subject to full-text review. After detailed reviewing, 29 studies were excluded. Of these 29 excluded studies, four VH-IVUS studies [17–20] did not provide the absolute volume of plaque composition and were excluded. In total, ten studies were included in the present meta-analysis (Fig 1) [8–10,15,21–26].

Of the ten included studies, five were RCTs [8,10,15,23,25] and five were nonrandomized cohort studies [9,21,22,24,26]. The baseline characteristics of the included studies are shown in Table 1. These ten studies enrolled a total of 682 patients and included 14 groups receiving statin treatment. The average age of the patients ranged from 54 to 65 years and 56%–90% were male. The mean LDL-C level was 119.1±32.9 mg/dL (range, 94.4–144.9 mg/dL) at baseline, and 75.3±25.5 mg/dL (range, 52.1–100.5 mg/dL) at follow-up. The length of follow-up ranged from 6 to 24 months. Rosuvastatin was used in four studies, atorvastatin in three studies, simvastatin in two studies, pitavastatin in one study, and fluvastatin in one study. Various types of statins were used in a single group in two studies [23,26]. The HEAVEN study [23] compared statins with other lipid-lowering agents plus statins, and we only pooled the results of the statins group. As the LAMIS study [22] divided patients receiving the same dose of pitavastatin into two groups based on the level of high-sensitivity C-reactive protein reduction, we combined the two groups into a single group.

**Table 1:** Demographics and characteristics of the included studies.

Study	Year	Study design	Patients (n)	Intervention	Dose (mg/d)	Mean age (years)	Men n (%)	LDL-C (mg/dL) at baseline	LDL-C (mg/dL) at follow-up	Follow-up (month)
Nasu et al.	2009	Nonrandomized	40	Fluvastatin	60	63±10	32(80)	144.9±31.5	98.1±12.7	12
			40	Usual care	NA					
Hong et al.	2009	RCT	50	Simvastatin	20	58±10	40(80)	119±30	78±20	12
			50	Rosuvastatin	10	59±9	37(74)	116±28	64±21	12
HEAVEN	2012	RCT	47	Statins	NA	65.1±10.6	31(66)	104.4±30.9	100.5±30.9	12
			42	Statins+ezetimibe	NA					
Eshtehardi et al.	2012	Nonrandomized	20	Atorvastatin	80	54	13(65)	120.7±8.8	70.7±8.3	6
Shin et al.	2012	Nonrandomized	24	Simvastatin	20	60.2 ± 8.2	16 (73)	105.5±50.2	60.6±20.9	12
			24	Rosuvastatin	10	61.6 ± 9.3	13 (56)	94.4±30.6	53.4±21.4	12
VENUS	2012	RCT	19	Atorvastatin	10	65.05±9.99	14 (74)	122.39±39.54	68.53±26.80	6
			20	Atorvastatin	40	63.70±9.80	18 (90)	112.35±27.14	52.12±12.63	6
LAMIS	2012	Nonrandomized	94	Pitavastatin	2	64.3±10.1	68 (72)	118.9±29.7	85.9±20.8	8
Hwang et al.	2013	Nonrandomized	54	Statins	NA	59±10	38 (70)	119.7±31.4	67.3±20.4	6
VIRHISTAMI	2013	RCT	44	Rosuvastatin	5	60.0±10.3	38(86.4)	119.9±27.1	77.3±15.5	12
			43	Rosuvastatin	40	62.0±9.9	35(81.4)	119.9±38.7	61.9±27.1	12
Puri et al.	2014	RCT	71	Rosuvastatin/Atorvastatin	40/80	57.6±9.0	59(80.3)	128.6±30.7	72.4±25.9	24

Abbreviations: RCT: randomized controlled trial; LDL-C: low-density lipoprotein cholesterol.

### Risk of bias

Among the five included RCTs, three studies showed a low risk of bias, one study was unclear, and one study showed a high risk of bias as the outcome assessment was not blinded (S2 Table). According to the Newcastle–Ottawa scale for cohort studies, all nonrandomized cohort studies were rated as high quality (S3 Table).

### Effect of statins on plaque composition

**Fig 2:**
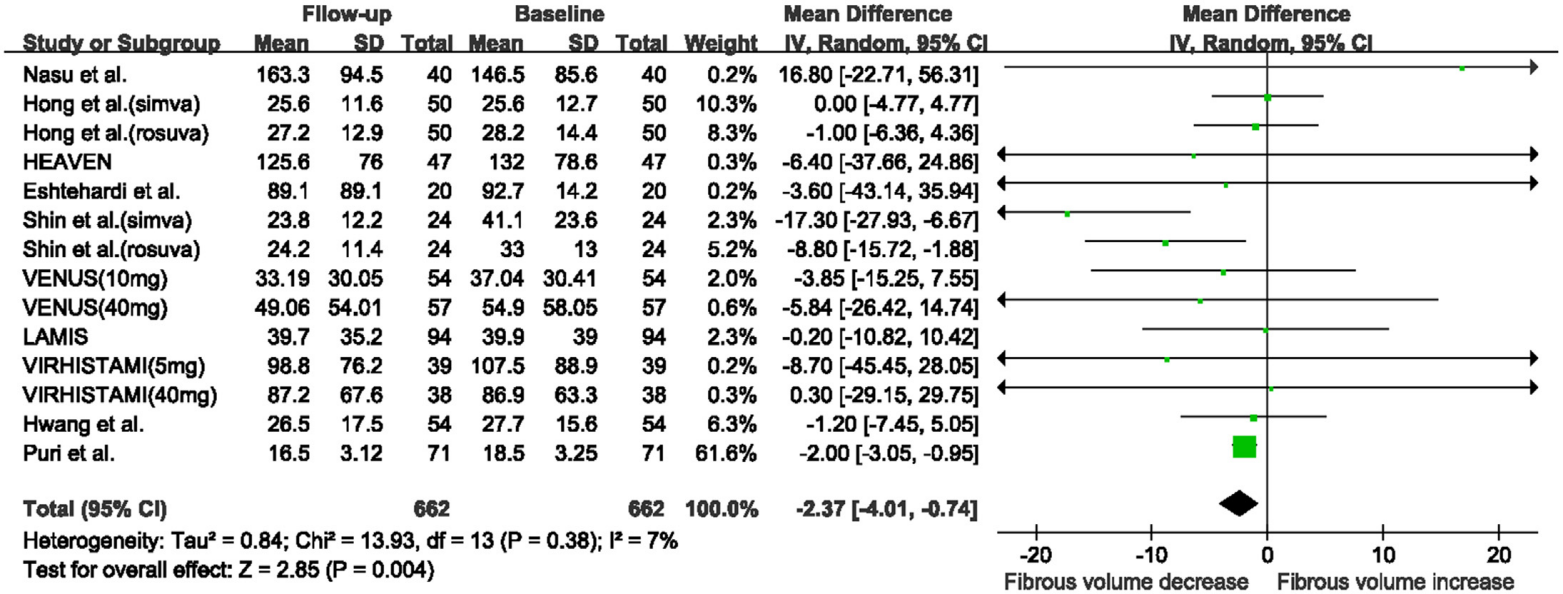
Changes in fibrous volume between follow-up and baseline with statin therapy. Abbreviations: CI: confidence interval; SD: standard deviation.

**Fig 3:**
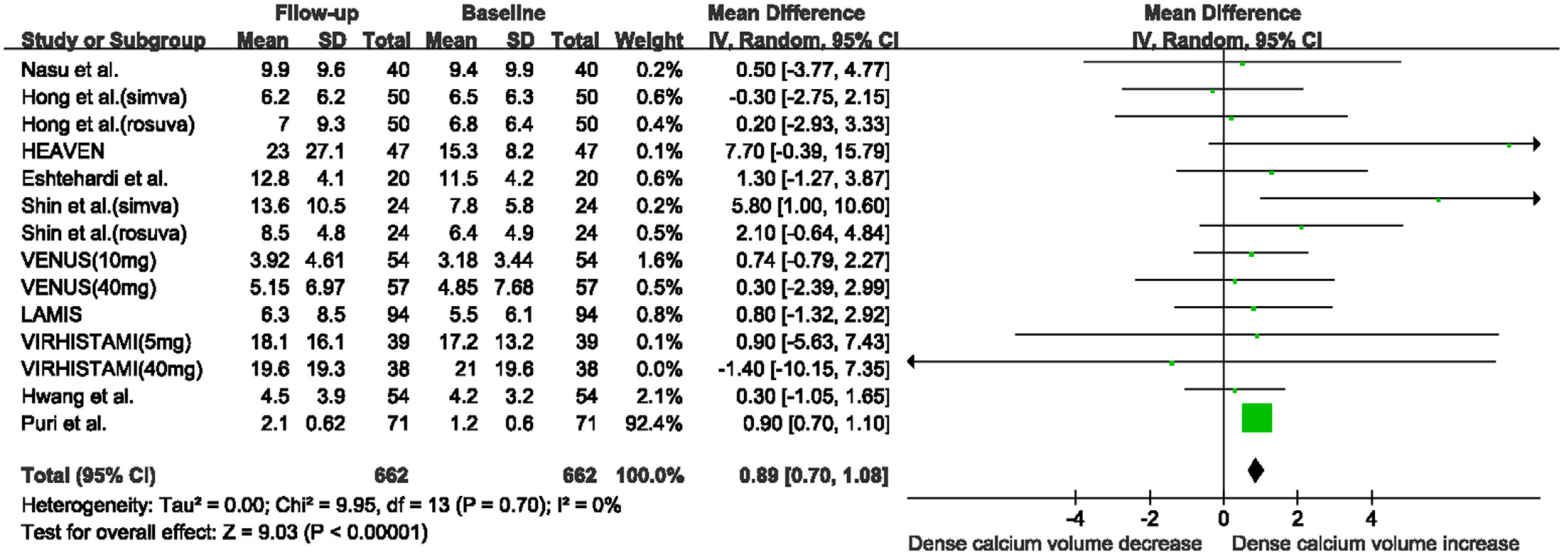
Changes in dense calcium volume between follow-up and baseline with statin therapy. Abbreviations: CI: confidence interval; SD: standard deviation.

There was a marked reduction in fibrous volume between baseline and follow-up (WMD: -2.37 mm^3^, 95% CI -4.01 to -0.74 mm^3^, I^2^ = 7%, P = 0.004; Fig 2), and a significant increase in DC volume (WMD: 0.89 mm^3^, 95% CI 0.70 to 1.08 mm^3^, I^2^ = 0%, P<0.00001, Fig 3). No significant change was seen in FF volume (WMD: -2.00 mm^3^, 95% CI -5.84 to 1.84 mm^3^, I^2^ = 85%, P = 0.31; S1 Fig) and NC volume (WMD: 0.11 mm^3^, 95% CI -0.39 to 0.60 mm^3^, I^2^ = 0, P = 0.67; S2 Fig). The funnel plots for the NC and DC volume analyses revealed no evidence of publication bias (S3A and S3B Fig). The funnel plots for the fibrous and FF volume analyses appeared asymmetric (S4A and S4B Fig). However, Egger’s test suggested a low probability of publication bias (P = 0.42 and P = 0.07, respectively).

**Fig 4:**
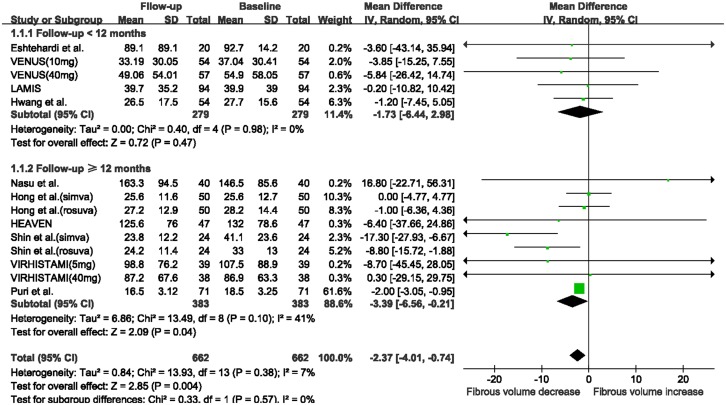
Meta-analysis of changes in fibrous volume with statin therapy using stratified analysis based on follow-up duration. Abbreviations: CI: confidence interval; SD: standard deviation.

**Fig 5:**
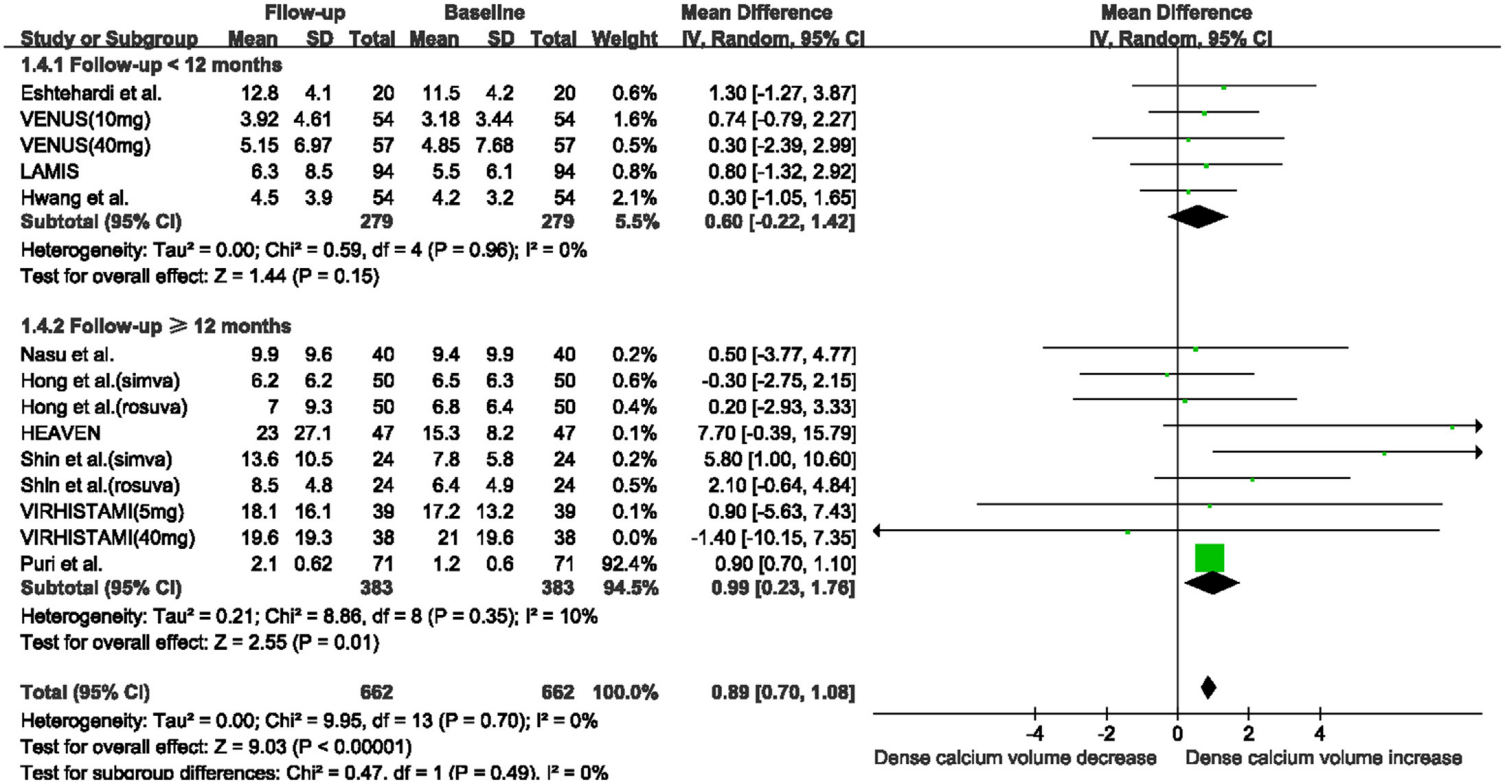
Meta-analysis of changes in dense calcium volume with statin therapy using stratified analysis based on follow-up duration. Abbreviations: CI: confidence interval; SD: standard deviation.

When stratifying studies according to follow-up duration, the fibrous volume was substantially decreased in the subgroup with ≥12 months follow-up (WMD: -3.39 mm^3^, 95% CI -6.56 to -0.21 mm^3^, I^2^ = 41%, P = 0.04; Fig 4), while no significant change was observed in the subgroup with < 12 months follow-up (WMD: -1.73 mm^3^, 95% CI -6.44 to 2.98 mm^3^, I^2^ = 0, P = 0.47). The absolute DC volume was also significantly increased in the subgroup with ≥12 months follow-up (WMD: 0.99 mm^3^, 95% CI 0.23 to 1.76 mm^3^, I^2^ = 10%, P = 0.01; Fig 5), while no change was observed in the subgroup with < 12 months follow-up (WMD: 0.60 mm^3^, 95% CI -0.22 to 1.42 mm^3^, I^2^ = 0%, P = 0.15). There were no substantial changes in FF and NC volumes in the two subgroups after statin therapy (S5A and S5B Fig).

**Fig 6:**
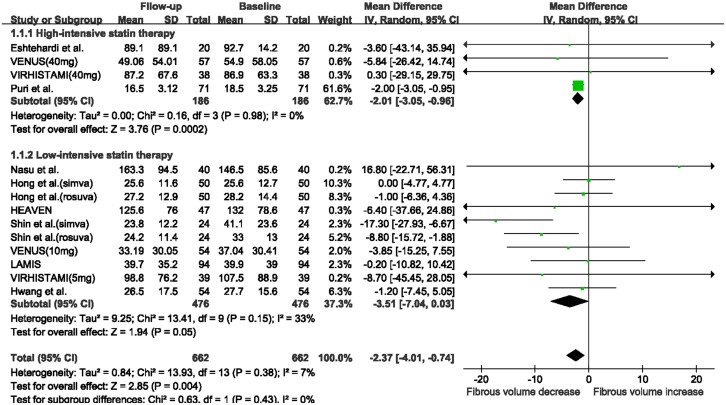
Meta-analysis of changes in fibrous volume with statin therapy using stratified analysis based on the intensity of statin therapy. Abbreviations: CI: confidence interval; SD: standard deviation.

**Fig 7:**
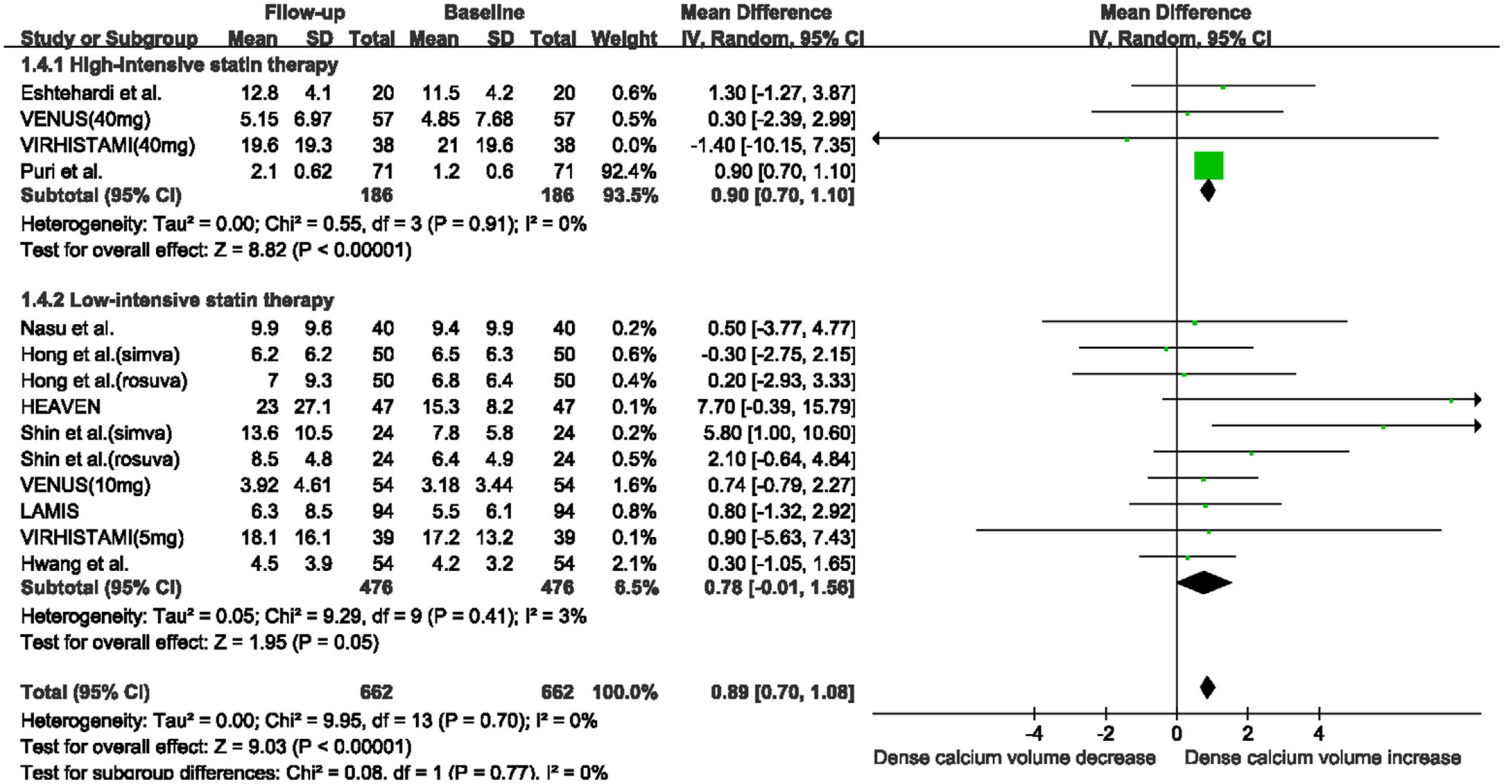
Meta-analysis of changes in dense calcium volume with statin therapy using stratified analysis based on the intensity of statin therapy. Abbreviations: CI: confidence interval; SD: standard deviation.

When stratifying studies according to the intensity of statin therapy, a significant reduction in fibrous volume was seen in the high-intensive statin therapy subgroup (WMD: -2.01 mm^3^, 95% CI -3.05 to -0.96 mm^3^, I^2^ = 0%, P = 0.0002; Fig 6), while the reduction in fibrous volume in the low-intensive statin therapy subgroup approached statistical significance (WMD: -3.51 mm^3^, 95% CI -7.04 to 0.03 mm^3^, I^2^ = 33%, P = 0.05). In addition, a substantial increase in DC volume was observed in the high-intensive statin therapy subgroup (WMD: 0.90 mm^3^, 95% CI 0.70 to 1.10 mm^3^, I^2^ = 0%, P< 0.00001; Fig 7), while the increase in absolute DC volume in the low-intensive statin therapy subgroup approached statistical significance (WMD: 0.78 mm^3^, 95% CI -0.01 to 1.56 mm^3^, I^2^ = 3%, P = 0.05). There was no significant change in FF and NC volumes in these two subgroups after statin treatment (S6A and S6B Fig).

Taking all plaque compositions together, the total plaque volume tended to decrease after statin treatment (WMD: -4.69 mm^3^, 95% CI -9.57 to 0.18mm^3^, I^2^ = 0%, P = 0.06; S7 Fig). The funnel plots for the plaque volume analysis revealed no evidence of publication bias (S8 Fig).

## Discussion

In the present meta-analysis, statin therapy not only tended to reduce plaque volume, but also significantly modified plaque composition including a significant reduction in fibrous volume and an increase in DC volume, whereas no significant changes in FF and NC volumes were observed. In stratified analyses, this modification in plaque composition was only seen in the subgroups with ≥12 months treatment duration or high-intensive statin therapy.

It is widely recognized that statin therapy is associated with reduction in coronary plaque volume as also shown in our study. However, the effect of statin therapy on plaque composition remains controversial. Hong and his colleagues suggested that 12 months of statin treatment was related to a significant increase in FF volume and an overt decrease in NC volume in patients without significant lesion stenosis [8]. Conversely, Shin et al. reported that there were significant decreases in fibrous and FF volumes and an overall increase in DC volume after one year of statin treatment, while no significant change in NC volume was seen [24]. In the HEAVEN study, atorvastatin was associated with decreases in fibrous and FF tissues and an increase in calcified tissue [23]. After pooling these studies, we found a significant increase in DC volume and a substantial decrease in fibrous volume. Compared with a previous meta-analysis [7], which only included two studies, eight new trials were added in the present study. Therefore, the statistical power was strengthened and the results were more conclusive.

Thin-cap fibroatheroma (TCFA), referred to as a vulnerable or unstable plaque, is characterized by a large NC and an overlying thin fibrous cap [27]. Unfortunately, none of the studies included evaluated whether statins decreased the amount of TCFA. This makes it difficult to understand the clinical significance of decreased fibrous volume due to statin therapy. Undoubtedly, the amount of fibrous tissue is not equal to the thickness of the fibrous cap. The spatial configuration of different compositions may be similar to or even more important than the amount. Indeed clinical studies have shown that statin therapy induced greater fibrous cap thickness in coronary plaques [28,29]. Decreased fibrous volume may result in decreased total plaque volume [30,31]. To date, most clinical trials included in the composition analysis were pre-post comparisons of statin therapy rather than placebo-controlled. This pre-post therapy comparison without placebo control makes it difficult to follow the natural progression of plaque composition. Although statin therapy did not reduce necrotic core, it may at least inhibit necrotic progression. Indeed, in the study by Nasu et al., which was a unique placebo-controlled study and included in the present meta-analysis, it was found that although fluvastatin did not induce a significant change in NC volume compared with baseline, a significant reduction in NC volume was seen in the fluvastatin group compared with the placebo group [9].

It is worth noting that plaque calcification increased after statin treatment. This result was consistent with previous studies using calcium scoring, which have shown that statin treatment was associated with coronary calcium progression [32,33]. Controversy exists regarding whether calcification is good or bad. Some studies found that calcification was more common in plaques of stable angina than in acute coronary syndromes [34], and considered calcification a contributor to the stabilization of plaques [35]. On the other hand, other studies showed that calcification indicated the advancement of atherosclerosis and was associated with poorer outcomes [36,37]. Some evidence has suggested that microcalcifications and calcified nodules may play an important role in atherosclerotic plaque vulnerability [38,39]. Microcalcification induces inflammation and promotes plaque rupture, whereas macrocalcification which refers to the sheet-like appearance of calcification acts as a barrier to inflammation and stabilizes the plaque [40,41]. Based on the above results, it seems rational to deduce that the pattern and location rather than the amount of calcification contribute to atherosclerotic plaque progression [15].

Evidence from clinical trials show that long-term and intensive statin therapy was beneficial in slowing the progression of atherosclerotic plaques than short-term and low statin therapy [6,7,42,43]. Similarly, our stratified study indicated that ≥12 months and high-intensive statin therapy, rather than <12 months and low-intensive statin therapy, promoted overt modification of plaque composition. These results may provide strong support for long-term intensive statin therapy in coronary atherosclerotic diseases.

Our analysis had some limitations. Firstly, we pooled data from different studies with different population demographics, severity of illness, length of follow-up and different doses and types of statins. These discrepancies among studies may have contributed to the presence of high heterogeneity between studies in some of our analyses. Secondly, because the study by Puri et al. [15] provided data on median and range, we estimated the mean and SD of the data, which may have led to potential deviation of the results. Nevertheless, estimates of the mean and SD were fairly accurate and using the estimates was superior to excluding the trials [16]. Thirdly, there is a limitation in volume measurements since plaque volume may be influenced by the variability in lesion length [44]. In some IVUS studies, normalized or percentage plaque volume was used to compensate for this limitation [17,45]. However, even percentage plaque volume is influenced by vessel remodeling [1]. Further studies are warranted to determine a better indicator for plaque progression. Fourthly, the longitudinal distribution of coronary plaque composition plays an important role in plaque vulnerability [46]. However, no studies included provided the data of longitudinal change in plaque composition after statin therapy. Thus the impact of longitudinal change in plaque composition should be considered when assessing the effect of statin therapy on plaque composition. Finally, five included trials were nonrandomized studies with a potential risk of bias. However, all except one study were rated as high quality based on the Newcastle–Ottawa scale. In the future, more double-blind RCTs of IVUS should be emerging.

In conclusion, statin treatment, especially of high-intensity and long-term duration, induced a marked modification in coronary plaque composition including a significant decrease in fibrous volume and an increase in DC volume.

## Supporting Information

S1 Checklist PRISMA checklist. (DOC)

S1 Fig Changes in fibro-fatty volume between follow-up and baseline with statin therapy. (TIF)

S2 Fig Changes in necrotic core volume between follow-up and baseline with statin therapy. (TIF)

S3 Fig A: Funnel plot for the meta-analysis of necrotic core volume with statin therapy; B: Funnel plot for the meta-analysis of dense calcium volume with statin therapy. (TIF)

S4 Fig A: Funnel plot for the meta-analysis of fibrous volume with statin therapy; B: Funnel plot for the meta-analysis of fibro-fatty volume with statin therapy. (TIF)

S5 Fig A: Meta-analysis of changes in fibro-fatty volume with statin therapy using stratified analysis based on follow-up duration; B: Meta-analysis of changes in necrotic core volume with statin therapy using stratified analysis based on follow-up duration. (TIF)

S6 Fig A: Meta-analysis of changes in fibro-fatty volume with statin therapy using stratified analysis based on the intensity of statin therapy; B: Meta-analysis of changes in necrotic core volume with statin therapy using stratified analysis based on the intensity of statin therapy. (TIF)

S7 Fig Changes in plaque volume between follow-up and baseline with statin therapy. (TIF)

S8 Fig Funnel plot for the meta-analysis of plaque volume with statin therapy. (TIF)

S1 Table Search strategy for online electronic databases. (DOCX)

S2 Table Risk of bias assessments for the included randomized controlled trials. (DOCX)

S3 Table Risk of bias for the included nonrandomized studies. (DOCX)
