# Supplementary figures and images for: The Effect of Statin Therapy on Coronary Plaque Composition Using Virtual Histology Intravascular Ultrasound: A Meta-Analysis
Source: PLoS One. 2015 Jul 30;10(7):e0133433. doi: 10.1371/journal.pone.0133433 (PMC4520465; doi:10.1371/journal.pone.0133433)

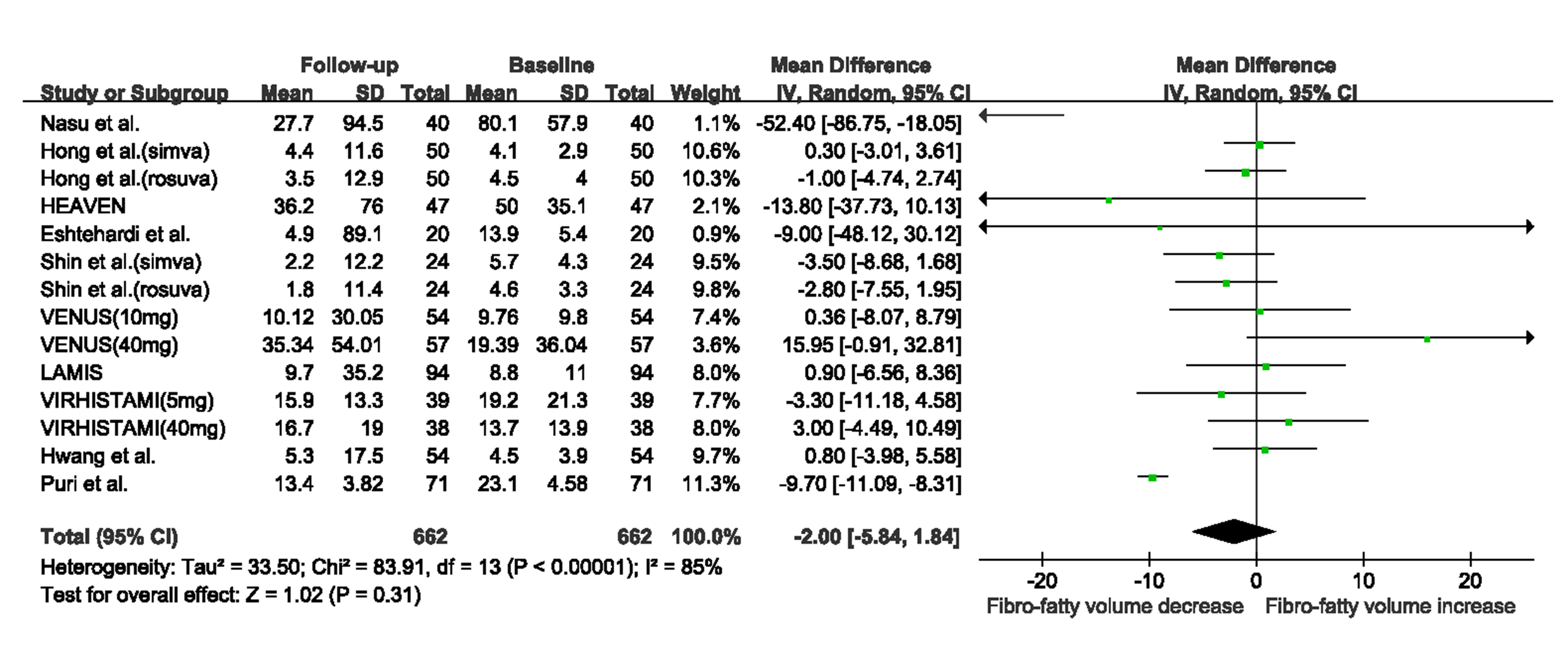

Supplement: S1 Fig — (TIF) [file pone.0133433.s002.tif]

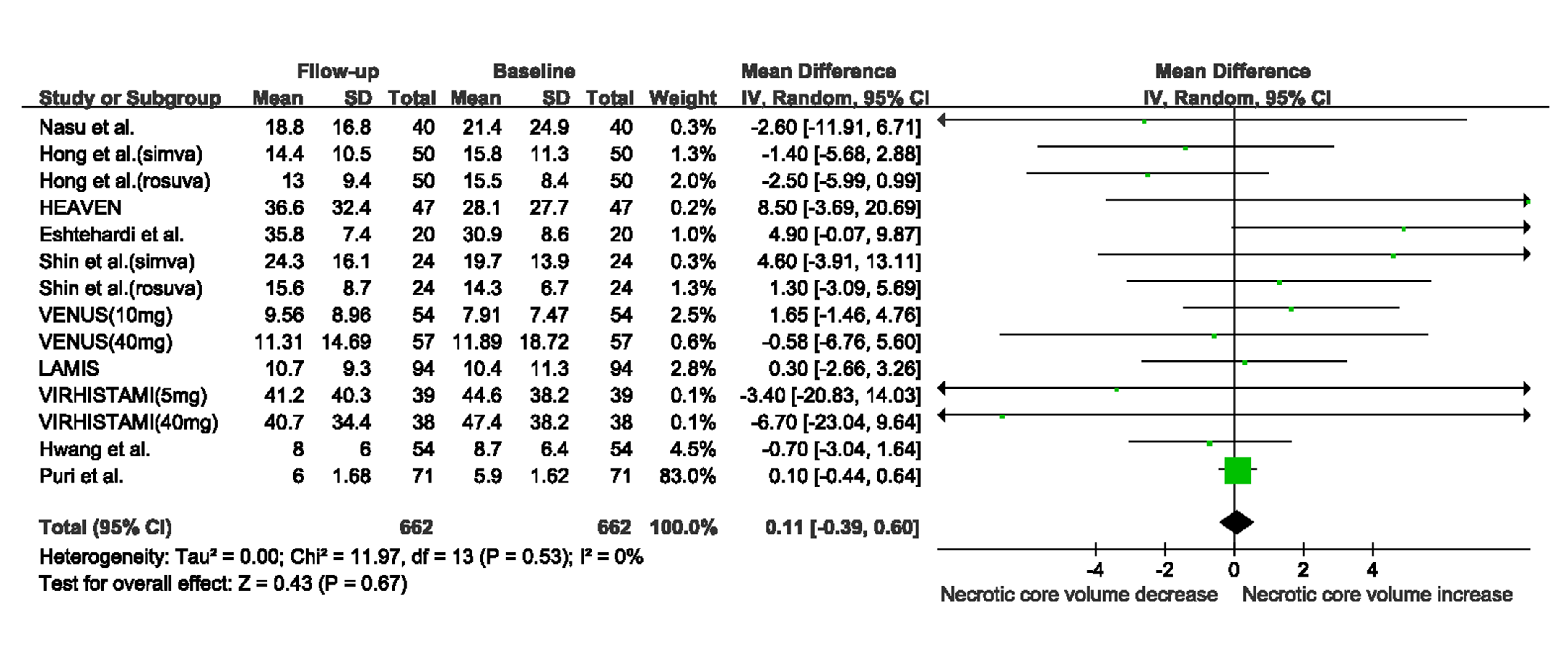

Supplement: S2 Fig — (TIF) [file pone.0133433.s003.tif]

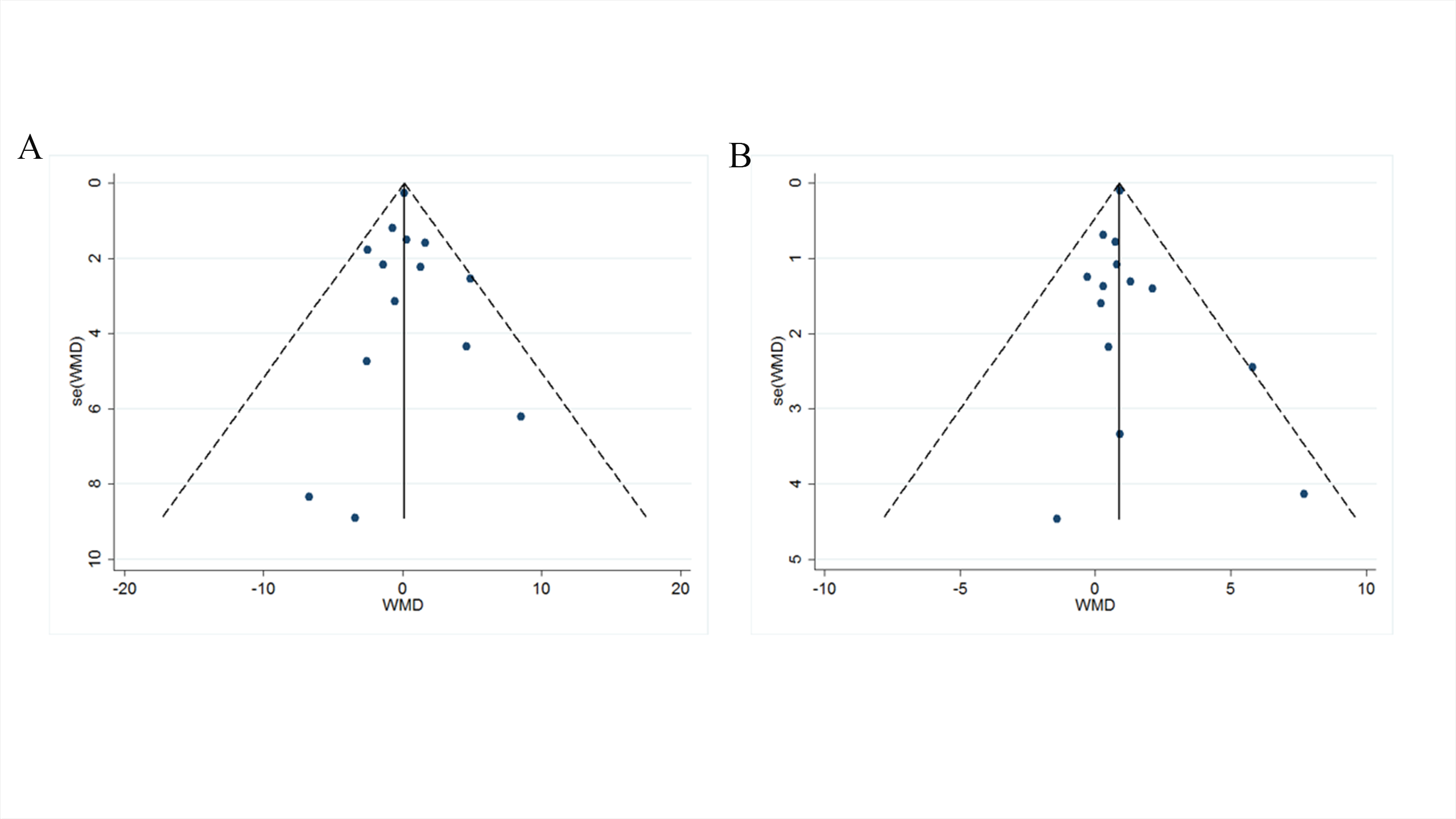

Supplement: S3 Fig — (TIF) [file pone.0133433.s004.TIF]

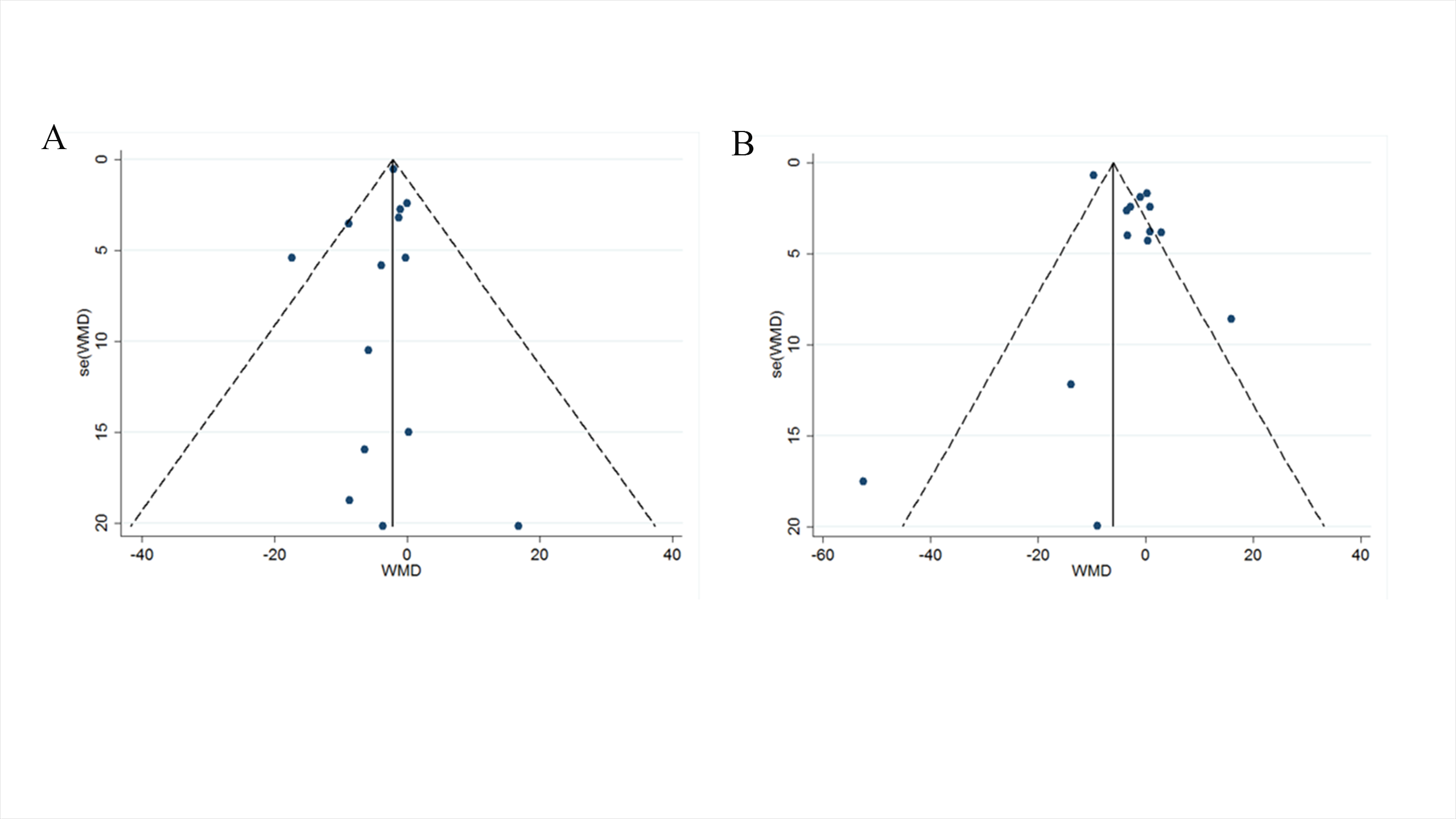

Supplement: S4 Fig — (TIF) [file pone.0133433.s005.TIF]

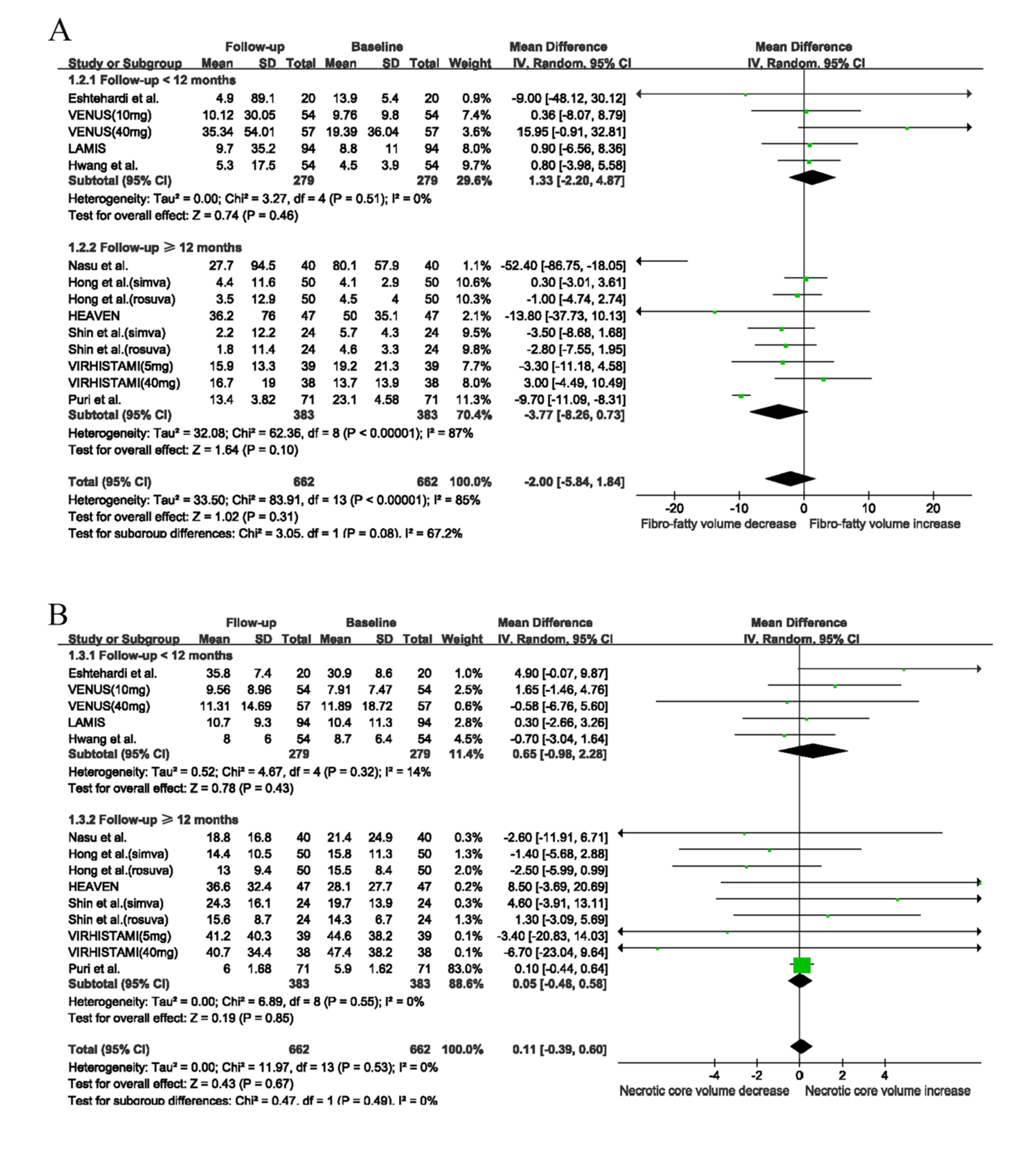

Supplement: S5 Fig — (TIF) [file pone.0133433.s006.tif]

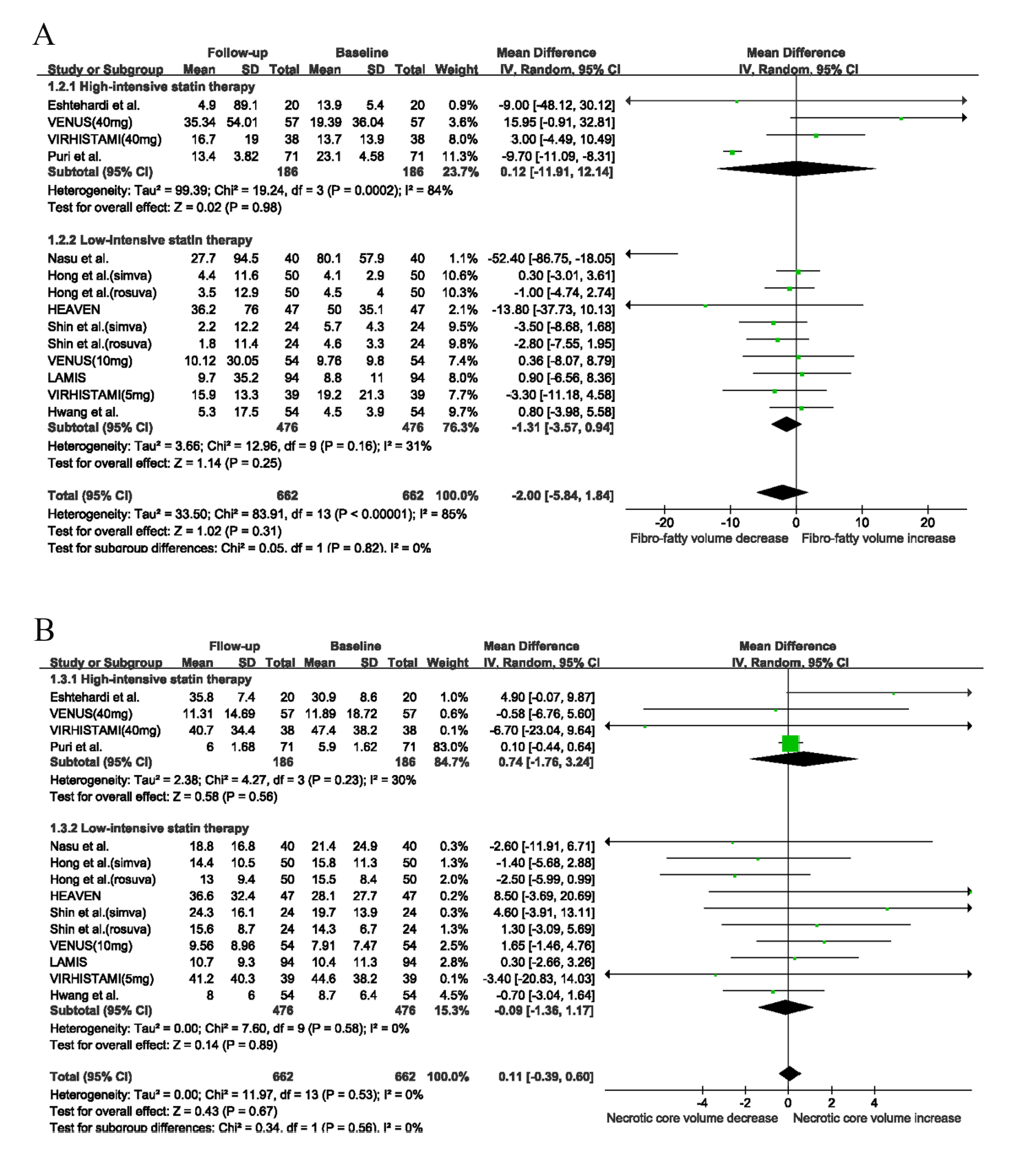

Supplement: S6 Fig — (TIF) [file pone.0133433.s007.tif]

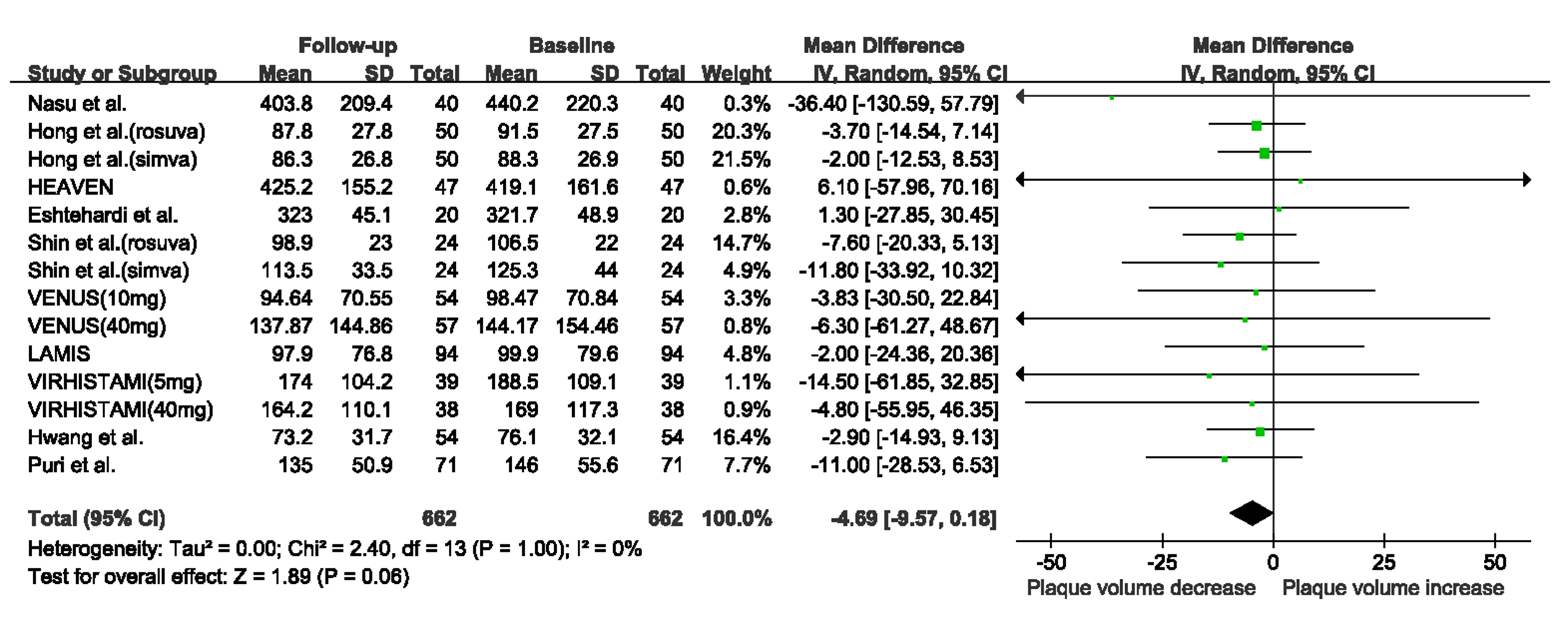

Supplement: S7 Fig — (TIF) [file pone.0133433.s008.tif]

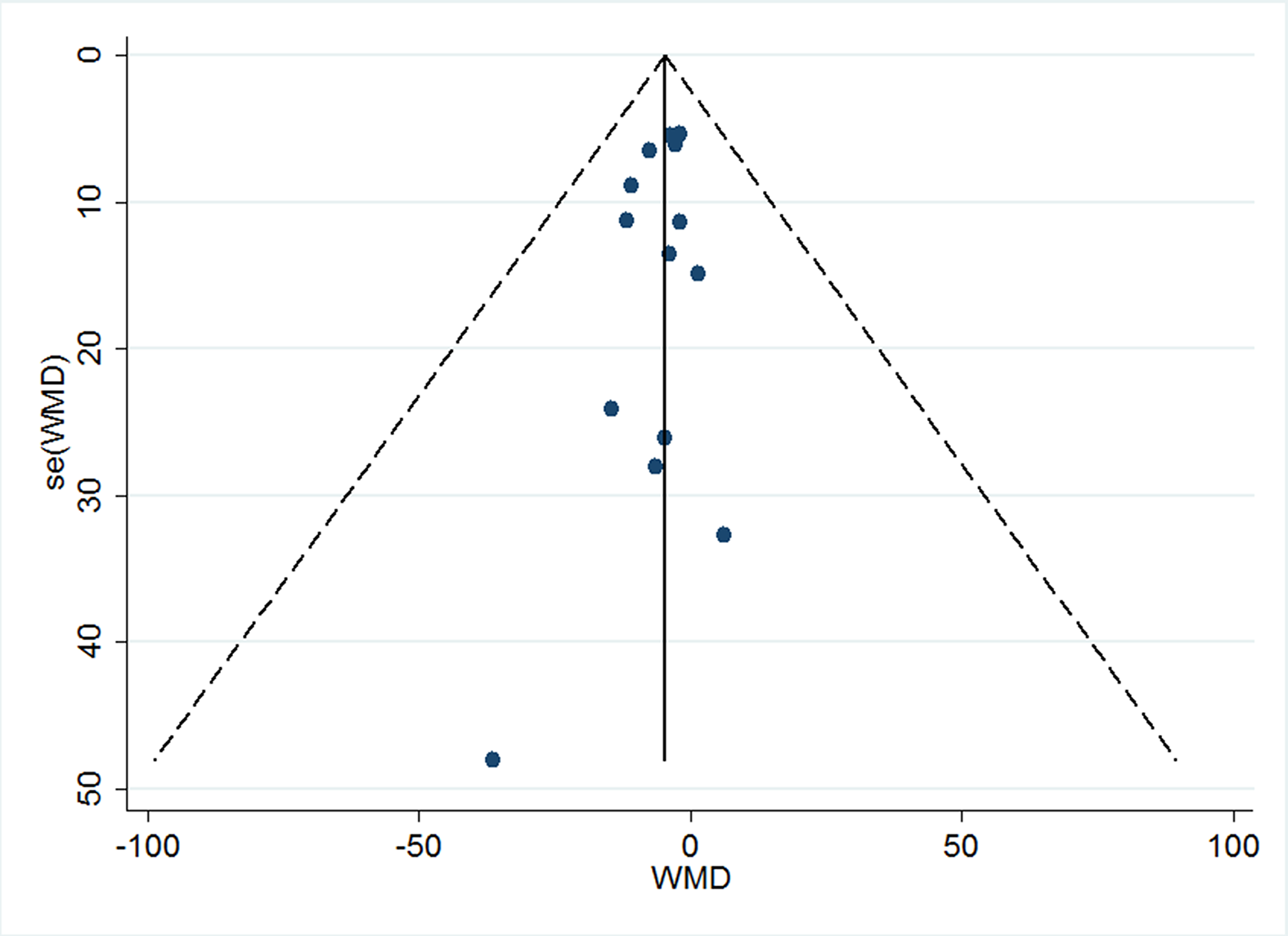

Supplement: S8 Fig — (TIF) [file pone.0133433.s009.tif]
