# Supplementary material for: The Effect of Statin Therapy on Coronary Plaque Composition Using Virtual Histology Intravascular Ultrasound: A Meta-Analysis
Source: PLoS One. 2015 Jul 30;10(7):e0133433. doi: 10.1371/journal.pone.0133433 (PMC4520465; doi:10.1371/journal.pone.0133433)
Supplement: S1 Table — (DOCX) [file pone.0133433.s010.docx]

| **Database** | **Search Strategy** |
| --- | --- |
| Pubmed | #1 ((((((((((((((statin[Title/Abstract]) OR statins[Title/Abstract]) OR HMG-CoA reductase inhibitors[Title/Abstract]) OR HMG-CoA reductase inhibitor[Title/Abstract]) OR 3-hydroxy-3-methylglutaryl coenzyme a reductase[Title/Abstract]) OR 3-hydroxy-3-methylglutaryl coenzyme a reductases[Title/Abstract]) OR atorvastatin[Title/Abstract]) OR pravastatin[Title/Abstract]) OR simvastatin[Title/Abstract]) OR cerivastatin[Title/Abstract]) OR fluvastatin[Title/Abstract]) OR lovastatin[Title/Abstract]) OR mevastatin[Title/Abstract]) OR pitavastatin[Title/Abstract]) OR rosuvastatin[Title/Abstract]  #2 ((Intravascular ultrasound[Title/Abstract]) OR intravascular ultrasonography[Title/Abstract]) OR IVUS[Title/Abstract]  #3 #1 AND #2 |
| Embase | #1 'HMG-CoA reductase inhibitors':ab,ti OR 'HMG-CoA reductase inhibitor':ab,ti OR '3-hydroxy-3-methylglutaryl coenzyme a reductase inhibitors':ab,ti OR '3-hydroxy-3-methylglutaryl coenzyme a reductase inhibitor':ab,ti OR statin:ab,ti OR statins:ab,ti OR atorvastatin:ab,ti OR pravastatin:ab,ti OR simvastatin:ab,ti OR cerivastatin:ab,ti OR fluvastatin:ab,ti OR lovastatin:ab,ti OR mevastatin:ab,ti OR pitavastatin:ab,ti OR rosuvastatin:ab,ti AND ([embase]/lim OR [embase classic]/lim)  #2 'intravascular ultrasound':ab,ti OR 'intravascular ultrasonography':ab,ti OR ivus:ab,ti AND ([embase]/lim OR [embase classic]/lim)  #3 #1 AND #2 |
| Web of science | #1 topic: (“HMG-CoA reductase inhibitors”) OR topic : (“HMG-CoA reductase inhibitor”) OR topic : (statin) OR topic: (statins) OR topic: ("3-hydroxy-3-methylglutaryl coenzyme a reductase inhibitors") OR topic: ("3-hydroxy-3-methylglutaryl coenzyme a reductase inhibitor") OR topic: (atorvastatin) OR topic: (simvastatin) OR topic: (pravastatin) OR topic: (cerivastatin) OR topic: (fluvastatin) OR topic: (lovastatin) OR topic: (mevastatin) OR topic: (pitavastatin) OR topic: (rosuvastatin)  #2 topic: (Intravascular ultrasound) OR topic : (IVUS) OR (intravascular ultrasonography)  #3 #1 AND #2 |
| Cochrane | #1 "HMG-CoA reductase inhibitors":ti,ab,kw or "HMG-CoA reductase inhibitor":ti,ab,kw or statin:ti,ab,kw or statins:ti,ab,kw or "3-hydroxy-3-methylglutaryl coenzyme a reductase inhibitor":ti,ab,kw or "3-hydroxy-3-methylglutaryl coenzyme a reductase inhibitors":ti,ab,kw or atorvastatin:ti,ab,kw OR pravastatin:ti,ab,kw OR simvastatin:ti,ab,kw OR cerivastatin:ti,ab,kw OR fluvastatin:ti,ab,kw OR lovastatin:ti,ab,kw OR mevastatin:ti,ab,kw OR pitavastatin:ti,ab,kw OR rosuvastatin:ti,ab,kw  #2 "intravascular ultrasound":ti,ab,kw or "intravascular ultrasonography":ti,ab,kw or ivus:ti,ab,kw  #3 #1 AND #2 |

**Table S1. Search strategy for online electronic databases.**
