# Supplementary material for: The Effect of Statin Therapy on Coronary Plaque Composition Using Virtual Histology Intravascular Ultrasound: A Meta-Analysis
Source: PLoS One. 2015 Jul 30;10(7):e0133433. doi: 10.1371/journal.pone.0133433 (PMC4520465; doi:10.1371/journal.pone.0133433)
Supplement: S2 Table — (DOCX) [file pone.0133433.s011.docx]

| **Study Name** | **Sequence generation** | **Allocation concealment** | **Blinding of participants, personnel and outcome assessors** | **Incomplete outcome data** | **Selective outcome reporting** | **Other sources of bias** |
| --- | --- | --- | --- | --- | --- | --- |
| Hong et al. 2009 | unclear | unclear | high | low | low | low |
| HEAVEN, 2012 | low | low | low | low | low | low |
| VENUS, 2012 | low | low | low | low | low | low |
| VIRHISTAMI,2013 | unclear | low | low | low | low | low |
| Puri et al. 2014 | low | low | low | low | low | low |

**Table S2. Risk of bias assessments for the included randomized controlled trials.**

The Hong et al.’s study was regarded as high risk of bias because of no blinded outcome assessment.
