# Supplementary material for: The Effect of Statin Therapy on Coronary Plaque Composition Using Virtual Histology Intravascular Ultrasound: A Meta-Analysis
Source: PLoS One. 2015 Jul 30;10(7):e0133433. doi: 10.1371/journal.pone.0133433 (PMC4520465; doi:10.1371/journal.pone.0133433)
Supplement: S3 Table — (DOCX) [file pone.0133433.s012.docx]

| **Study** | **Selection** | | | | **Comparability** | **Outcome** | | |
| --- | --- | --- | --- | --- | --- | --- | --- | --- |
|  | **Representativeness of the exposed cohort** | **Selection of the non exposed cohort** | **Ascertainment of exposure** | **Demonstration that outcome of interest was not present at start of study** | **Comparability of cohorts on the basis of the design or analysis** | **Assessment of outcome** | **Was follow-up long enough for outcomes to occur** | **Adequacy of follow up of cohorts** |
| Nasu et al. 2009 |  |  |  |  |  |  |  |  |
| Eshtehardi et al. 2012 |  |  |  |  |  |  |  |  |
| Shin et al. 2012 |  |  |  |  |  |  |  |  |
| LAMIS, 2012 |  |  |  |  |  |  |  |  |
| Hwang et al. 2013 |  |  |  |  |  |  |  |  |

**Table S3. Risk of bias for the included nonrandomized studies.**

According to Newcastle-Ottawa Scale for assessing the quality of nonrandomized studies, indicates one score. The maximum score on this scale is 9 points. A total score of

7-9 was defined as high quality, 4-6 as medium quality, and 1-3 as low quality. All nonrandomized studies had more than 7 scores and were regarded as high quality.
